# LLM-supported collaborative ontology design for data and knowledge management platforms

**DOI:** 10.3389/fdata.2025.1676477

**Published:** 2025-11-12

**Authors:** Janis Kampars, Guntis Mosans, Tushar Jogi, Franz Roters, Napat Vajragupta

**Affiliations:** 1 Information Technology Institute, Faculty of Computer Science, Information Technology and Energy, Riga, Latvia; 2 Microstructure Physics and Alloy Design, Max Planck Institute for Sustainable Materials, Düsseldorf, Germany; 3 VTT Technical Research Centre of Finland Ltd., Espoo, Finland

**Keywords:** ontology design, large language models, FAIR, experiment, simulation, hydrogen, metals

## Abstract

The management of vast, heterogeneous, and multidisciplinary data presents a critical challenge across scientific domains, hindering interoperability and slowing scientific progress. This paper addresses this challenge by presenting a pragmatic extension to the NeOn iterative ontology engineering framework, a well-established methodology for collaborative ontology design, which integrates Large Language Models (LLMs) to accelerate key tasks while retaining domain expert-in-the-loop validation. The methodology was applied within the HyWay project, an EU-funded research initiative on hydrogen-materials interactions, to develop the Hydrogen-Material Interaction Ontology (HMIO), a domain-specific ontology covering 29 experimental methods and 14 simulation types for assessing interactions between hydrogen and advanced metallic materials. A key result is the successful integration of the HMIO into a Data and Knowledge Management Platform (DKMP), where it drives the automated generation of data entry forms, ensuring that all captured data is Findable, Accessible, Interoperable, and Reusable (FAIR) and HMIO compliant by design. The validation of this approach demonstrates that this hybrid human-machine workflow for ontology engineering and further integration with the DKMP is an effective and efficient strategy for creating and operationalising complex scientific ontologies, thereby providing a scalable solution to advance data-driven research in materials science and other complex scientific domains.

## 1 Introduction

Contemporary research, particularly within complex multidisciplinary engineering domains (Suárez-Figueroa et al., 2015), increasingly produces large volumes of data marked by considerable heterogeneity. This diversity results from varying data formats, inconsistent terminologies, and limited interoperability across disciplines and tools. As a consequence, the resulting data landscape is often fragmented into isolated silos, where information generated in one experiment or simulation cannot be readily integrated with that from others. Such fragmentation poses significant challenges to knowledge discovery, collaboration, and the reproducibility of scientific results. These factors collectively highlight the need for advanced knowledge management methods, such as ontologies and specialized data platforms, to support the clear representation, effective sharing, and seamless integration of information.

This challenge is particularly relevant in critical research areas, such as the development of advanced materials and technologies for hydrogen, which are integral to supporting the European Union's strategic green energy transition and its overarching goal of achieving climate neutrality by 2050 (European Commission, 2020).

Semantic web technologies, particularly ontologies, offer a formal and shared conceptual framework that facilitates semantic interoperability, thereby addressing the challenge of data integration and supporting the implementation of the Findable, Accessible, Interoperable, and Reusable (FAIR) principles (Guizzardi, 2020). However, the development of high-quality, comprehensive ontologies is a well-recognized bottleneck. Traditional ontology engineering is a resource-intensive process that relies on the close collaboration of domain experts and ontology engineers, requiring both deep subject knowledge and specialized technical expertise; however, such approaches often face limitations in accommodating the evolving nature of scientific domains, as they lack effective support for continuous modification, maintenance, and reuse, which are essential factors for keeping ontologies up to date and broadly applicable (Spoladore and Pessot, 2021).

The emergence of Large Language Models (LLMs) provides a transformative approach to enduring challenges in ontology engineering, with the potential to significantly reduce the manual effort and time required for ontology development. Owing to their advanced natural language understanding and generation capabilities, LLMs hold considerable potential for automating and expediting key tasks in the early stages of ontology development. These tasks include the generation of competency questions for requirements specification, the extraction and conceptualization of ontological structures from source texts and terminologies, and support for ontology reuse through concept alignment and taxonomy matching (Garijo Verdejo et al., 2024).

This paper presents and validates a practical methodology that extends the NeOn iterative framework by integrating LLM-based automation as a co-pilot within a collaborative, expert-driven workflow. This socio-technical approach was developed and implemented within the HyWay project to create the Hydrogen-Material Interaction Ontology (HMIO) and integrate it into a Data and Knowledge Management Platform (DKMP). The primary contribution of this work is a practical, end-to-end demonstration of how this hybrid human-machine workflow can efficiently support the development of a complex, domain-specific ontology and facilitate the production of data that is inherently aligned with the FAIR principles. The subsequent sections detail this methodology, its technical implementation, the results achieved, and a critical discussion of its implications for the future of scientific data management.

## 2 Materials and methods

### 2.1 State of the art in ontology design and integration

#### 2.1.1 Methodologies for collaborative ontology design

Ontology has evolved into an engineering discipline, guided by established methodologies that provide systematic support throughout the design process. In complex and multidisciplinary research environments, especially those involving geographically distributed teams, it is essential to adopt approaches that promote both collaboration and flexibility. A variety of methodologies have been proposed, each emphasizing different phases and aspects of the ontology engineering lifecycle.

General strategies for conceptual design are typically grouped into three categories: top down, bottom up, and middle out. The top down approach begins with high-level abstract concepts and incrementally refines them into more specific ones. The bottom up approach derives general categories by analyzing concrete, domain-specific instances. The middle out approach starts with key mid-level concepts and extends them in both directions, capturing higher-level abstractions as well as more detailed, granular concepts, thereby aiming for a balanced and contextually relevant ontology structure.

UPON Lite (De Nicola and Missikoff, 2016) is a user-centric methodology designed to support rapid ontology engineering by enabling domain experts to lead the development process with minimal involvement from ontology engineers until the final formalization stage. The methodology comprises six structured steps: (1) constructing a domain-specific lexicon, (2) developing a glossary with definitions, (3) organizing terms into a hierarchical taxonomy based on subclass relations, (4) defining conceptual properties, (5) specifying part-whole relationships, and (6) encoding the resulting model using formal representation languages, typically OWL. UPON Lite adopts a participatory approach that promotes collaborative knowledge elicitation through shared, transparent tools such as online spreadsheets. Initially developed for enterprise and business applications, the methodology supports rapid prototyping and emphasizes the active role of end users to ensure the practical relevance and applicability of the resulting ontology. UPON Lite has been successfully applied in diverse domains including safety regulations, data science, intellectual property, smart building, and social networks, and is specifically demonstrated by De Lille and Roelens (2021) through a detailed case study in a public organization for internal audits.

MeLOn (Mockus and Palmirani, 2017) is an interdisciplinary, goal-oriented, and evaluation-driven methodology for ontology engineering, structured into ten clearly defined steps. It begins by establishing the ontology's objectives, identifying use cases for empirical testing, and formulating research questions. Simultaneously, it defines measurable evaluation criteria, including completeness, correctness, coherence, applicability, effectiveness, intuitiveness for non-specialists, computational soundness, and reusability. The methodology places strong emphasis on the analysis and reuse of existing ontological resources, ensuring that prior knowledge is integrated effectively. It guides the development of a domain-specific glossary to create a consistent conceptual foundation. Ontology construction is carried out using structured tabular representations that capture concepts, object and data properties, and formal constraints. These are then translated into UML diagrams and subsequently formalized in ontology languages such as OWL. MeLOn adopts an iterative development process that includes empirical scenario testing and systematic refinement based on evaluation outcomes. Domain experts play a central role in this cycle, which is repeated across multiple iterations and often supported by web-based interfaces to facilitate collaborative validation and feedback. The MeLOn methodology has been notably applied in the legal domain, including the development of PrOnto by Palmirani et al. (2018)—an ontology for the General Data Protection Regulation (GDPR) aimed at supporting legal reasoning and compliance verification.

DILIGENT (Pinto H. S. et al., 2004) is a fine-grained methodology designed to support the collaborative development and continuous evolution of ontologies in distributed environments. It was developed in response to the limitations of traditional centralized ontology engineering approaches, which are often unsuitable for scenarios involving dispersed stakeholders, substantial involvement of domain experts outside the core development team, and the need for sustained ontology evolution. The methodology comprises five key activities: the creation of an initial ontology, local adaptation by users, submission and evaluation of change requests by a control board, revision of the shared ontology, and subsequent updates at the local level. A defining characteristic of DILIGENT is its argumentation-based framework for managing consensus-building among contributors. It organizes exchanged arguments into types such as elaboration, justification, alternatives, examples, and counterexamples to facilitate structured discussion and decision-making. Validation efforts indicate that structured argumentation, when supported by appropriate collaboration mechanisms and tools, can significantly improve the efficiency and focus of ontology development in distributed settings. The DILIGENT methodology was applied by Pinto S. et al. (2004) in a knowledge sharing scenario among tourism stakeholders in the Balearic Islands, where organizations collaboratively developed and evolved a shared ontology to harmonize decentralized information about sustainability, technology, and hospitality.

The NeOn Methodology (Suárez-Figueroa et al., 2015) is a scenario-based and flexible framework for ontology engineering, distinguished by its emphasis on reusing and re-engineering existing knowledge resources and supporting dynamic, collaborative development. Rather than enforcing a fixed sequence of steps, NeOn provides a set of nine customisable scenarios that address diverse development contexts. These include building new ontologies from scratch, reusing and adapting non-ontological resources, reusing and merging existing ontologies, localizing ontologies, aligning multiple ontologies, and employing ontology design patterns to guide modeling decisions. A core strength of the NeOn Methodology lies in its support for both a traditional waterfall lifecycle model and an iterative and incremental lifecycle, designed for complex, large-scale, and evolving environments where stakeholder requirements and domain understanding develop over time. The methodology explicitly integrates activities for knowledge acquisition, conceptualization, formalization, implementation, maintenance, and validation, and emphasizes stakeholder involvement, quality assessment, and documentation throughout the ontology lifecycle. It also includes guidance for collaborative development settings, supporting asynchronous contributions and team coordination. The methodology has been validated in diverse application domains. Li et al. (2020) developed the Materials Design Ontology following several NeOn scenarios such as reuse of non-ontological resources and restructuring of ontological resources.

#### 2.1.2 The role of LLMs in ontology engineering

Large Language Models (LLMs) represent a significant advancement in ontology engineering by introducing data-driven, language-oriented artificial intelligence to support and accelerate tasks traditionally rooted in symbolic and logic-based approaches.

In the area of ontology generation from natural language, Lippolis et al. (2025) present and evaluate two prompting strategies, namely Memoryless CQbyCQ and Ontogenia, to automatically generate OWL ontologies from user stories and competency questions. Their study demonstrates that LLM-generated ontologies can match or even exceed the quality of those produced by novice engineers, especially when leveraging well-structured prompts and evaluation criteria such as logical consistency, completeness, and usability.

A second major direction is ontology construction from structured datasets, as exemplified by the OntoGenix workflow developed by Val-Calvo et al. (2025). This approach applies LLMs to derive ontological structures directly from commercial datasets, including entity definitions and RDF mappings. OntoGenix supports a configurable, modular pipeline for ontology planning and entity enhancement, achieving modeling results that closely approximate expert-crafted ontologies, albeit with some limitations in complex scenarios.

LLMs have also been investigated for ontology alignment and enhancement. Though not the central focus of the aforementioned works, studies by Babaei Giglou et al. (2023) and Fathallah et al. (2025) explore the use of LLMs to align new concepts with existing ontologies or to translate controlled natural language into formal axioms, demonstrating early but promising capabilities in supporting semantic interoperability and reuse.

While LLMs offer substantial promise for ontology engineering, their limitations are well documented. LLMs often lack the depth of knowledge required for accurate modeling in specialized scientific domains, which can lead to superficial or incorrect representations. Consequently, current research supports the role of LLMs as assistive technologies within expert-led workflows, where rigorous validation by domain specialists is essential to ensure the correctness and scientific reliability of the resulting ontologies (Li et al., 2024).

#### 2.1.3 Ontology-based data integration patterns

Ontologies demonstrate their full potential when embedded within data management platforms, where they support the structuring, annotation, and interpretation of scientific data. Ontology-Based Data Integration (OBDI) leverages ontologies to facilitate structured, semantically rich, and accessible interaction with heterogeneous data sources. OBDI addresses challenges such as fragmented database schemas, data redundancy, and limited accessibility for users without technical expertise. It achieves semantic interoperability by introducing an ontology layer that resides between the conceptual and physical data layers.

Four principal architectural patterns have been identified in the literature: Single Ontology, Multiple Ontologies, Hybrid, and Global-as-View (GAV). These approaches differ in how ontologies are structured and related to data sources and to one another. Comprehensive reviews of these models are provided by Wache et al. (2002), Corcho et al. (2020), Ekaputra et al. (2017), and Xiao et al. (2018).

The Single Ontology approach employs a unified global ontology to which all data sources are directly mapped. It offers conceptual simplicity and low implementation effort, particularly when data sources are semantically aligned. However, this architecture can be rigid, making it less suitable for integrating semantically diverse or evolving sources, and maintenance can become costly when changes occur in the system.

In contrast, the Multiple Ontologies model assigns a local ontology to each data source. Semantic interoperability is achieved through mappings between these local ontologies. This model excels in heterogeneous environments and allows independent ontology evolution, but it requires significant mapping effort, especially as the number of ontologies grows.

The Hybrid approach combines elements of both previous models by aligning local ontologies with a shared upper-level ontology or vocabulary. This strategy promotes interoperability while preserving domain-specific detail. It is particularly well-suited for multidisciplinary domains, where both semantic alignment and autonomy are necessary (Ekaputra et al., 2017).

The Global-as-View (GAV) approach allows for the preservation of independently developed local ontologies by defining mappings that transform local representations into instances of a global ontology. This architecture supports high levels of heterogeneity and complex mappings while retaining the benefits of both centralized and decentralized models.

These architectural models are not only theoretical constructs but have been realized in a variety of operational platforms designed to manage and integrate research data. The implementation of OBDI principles in real-world systems illustrates how semantic technologies can be used to support (Findable, Accessible, Interoperable, and Reusable (FAIR) data management.

For example, Coscine is a research data management (RDM) platform designed to support FAIR data principles by introducing a semantic interoperability layer over existing storage services (Lang et al., 2024). Its architecture is grounded in Semantic Web standards, including RDF, OWL, and SHACL, which are used to define flexible, schema-driven metadata application profiles. Each resource in Coscine is assigned a Persistent Identifier (PID), and all associated metadata is structured using standardized vocabularies and ontologies, thereby enhancing data findability and cross-project interoperability. The platform supports both the reuse and creation of domain-specific terminologies through integrated vocabulary services, and these metadata schemas can be created or extended by individual research projects according to their specific needs. This architectural design aligns with the Hybrid OBDI paradigm, wherein discipline-specific application profiles serve as local ontologies built upon a shared semantic foundation based on W3C standards. Rather than enforcing a single, rigid global schema, Coscine allows for heterogeneity and extensibility while preserving semantic compatibility across projects through adherence to a common metadata model.

openBIS is an extensible research data management platform that serves as a central hub for managing metadata, experimental data, and data provenance across diverse scientific workflows (Lang et al., 2024). It provides a configurable data model that links experimental outputs to biological samples, materials, and protocols through a hierarchical structure encompassing entities such as Data Spaces, Projects, Experiments, Samples, and Datasets. While its conceptual architecture relies on a central metadata schema, openBIS is designed to accommodate heterogeneous and evolving data types across domains by supporting user-defined types and annotations through its reflective metadata model. The system exposes comprehensive Application Programming Interfaces (APIs) and supports plugin-based extensibility, facilitating seamless integration with domain-specific analytical tools and enabling automation across the research data lifecycle. Provenance tracking is a core design feature, ensuring traceability of experimental processes and data transformations. Although openBIS employs a shared core schema, its capacity to flexibly represent heterogeneous data and integrate with external systems indicates that it aligns more closely with hybrid ontology-based data integration principles rather than a rigid single-ontology approach. Specifically, it maintains a shared semantic core while allowing local extensions and system-specific metadata configurations, thereby supporting both interoperability and domain-specific detail.

The NOMAD platform provides infrastructure for managing and sharing materials science data, initially focusing on computational simulations and more recently extending to experimental datasets (Scheidgen et al., 2023). It adopts a schema-driven architecture in which modular, code-specific parsers extract structured metadata from a wide range of simulation and instrument file formats. At its core is the NOMAD Metainfo (Ghiringhelli et al., 2023), a hierarchical and extensible metadata schema designed to capture both shared concepts across domains and specialized, tool-specific information. This flexibility supports semantic interoperability without enforcing a rigid global model, aligning with the hybrid ontology-based data integration approach. To facilitate metadata harmonization in experimental materials science, NOMAD integrates the NeXus ontology (Könnecke et al., 2015), a community-developed schema and data format built upon HDF5. NeXus provides a formal structure for describing experimental configurations, instrument settings, and measurement data, supporting machine readability and enabling data reuse across various facilities. The ontology component of NeXus, expressed in OWL, enhances compatibility with semantic web frameworks and extends its role as a standard reference model.

While all four OBDI patterns have distinct strengths, the hybrid approach is most frequently adopted in scientific data platforms. Its widespread use reflects a balance between semantic coherence, achieved through shared vocabularies or upper-level ontologies, and the flexibility needed to support heterogeneous data sources, evolving standards, and diverse project-specific needs.

### 2.2 The HyWay collaborative ontology design methodology

The Hyway methodology constitutes a practical, LLM augmented implementation of the NeOn ontology engineering life cycle. It adapts NeOn's scenario-based, iterative, and incremental framework by incorporating automation through large language models (LLMs), while customizing the initial knowledge acquisition strategy to reflect the specific context and needs of the project.

A foundational consideration in any ontology engineering project is the selection of appropriate knowledge sources. While large language models have demonstrated strong capabilities in ontology learning, particularly in extracting relevant concepts and relationships from unstructured textual sources such as scientific articles or technical documentation, this approach was not considered suitable for the HyWay project. The central limitation was the absence of a consolidated body of literature that accurately represented the domain-specific experimental and simulation workflows employed by the participating research partners. Building an ontology from generic literature risked misrepresenting the distinctive, practical aspects of these processes. To ensure fidelity to actual practice, the project adopted a direct expert-driven approach, surveying domain specialists to elicit relevant terminology, relationships, and parameterisations. This process produced a high-quality, expert-validated non-ontological resource that served as the foundation for subsequent stages.

**Figure 1:**
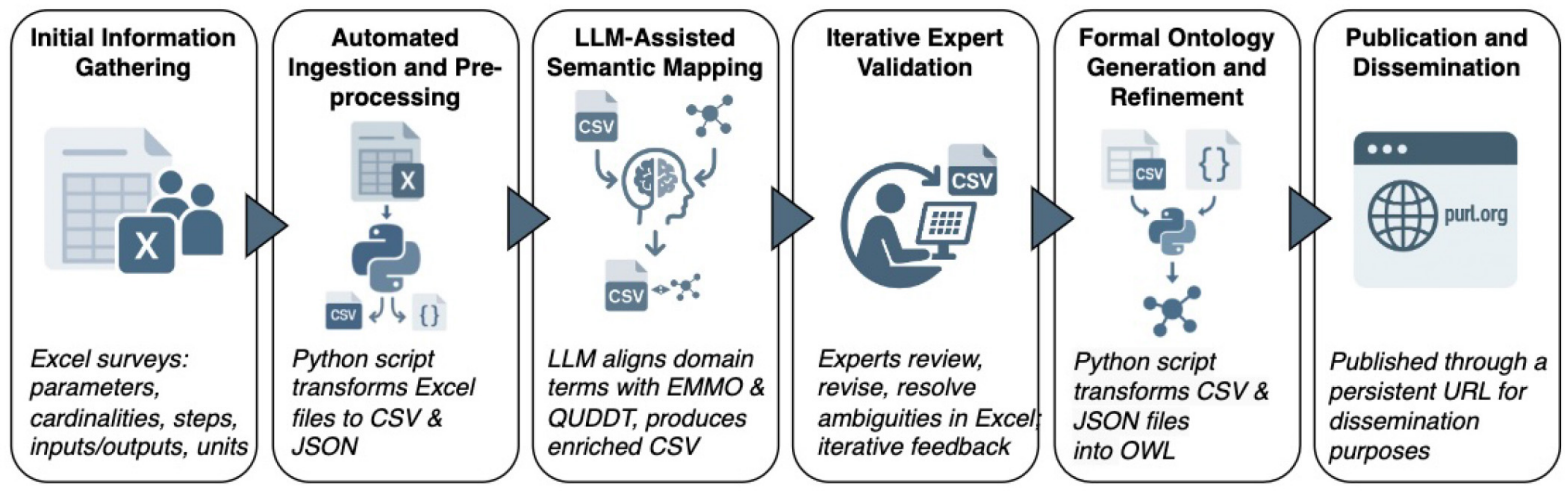
Steps of the HyWay methodology.

The Python application that supports this methodology is openly available (Kampars, 2025). As shown in Figure 1, the HyWay methodology proceeds through the following six stages:

1. Initial information gathering.

The process begins with the structured collection of domain-specific information from research partners. This is facilitated through a survey distributed as a Microsoft Excel template, which prompts users to document their experimental and simulation workflows. Respondents are asked to provide detailed information on process steps, parameters, input and output entities, cardinalities, and associated units of measurement. This stage establishes the foundational knowledge base upon which the ontology will be built.

To support and guide the initial schema design, the application profiles developed by the SFB 1394 Research Project (2023) were programmatically analyzed. These profiles describe experimental metadata schemas and were used to extract existing terms, which were subsequently integrated into the survey for review and completion by domain experts. However, it is important to note that not all experiments represented in HyWay align precisely with the schemas defined in SFB1394, and additional tailoring was required.

A list of metadata for the Düsseldorf Advanced Material Simulation Kit (DAMASK), one of the modeling suites used within the HyWay project, was prepared by systematically parsing the example input files provided in the package. The extracted parameters were categorized based on their association with DAMASK model variants plus those that are shared across models. Currently, five major model variants: *DAMASK_phenopowerlaw, DAMASK_isotropic, DAMASK_dislotwin, DAMASK_dislotungsten, DAMASK_nonlocal* are identified. The parameters common to all models were grouped under the label *DAMASK_Generic*. To support ontology generation, the parameters within each model variant were classified as *input* and/or *output*, based on their role in the simulation workflow. Additionally, the parameters were mapped to human-readable descriptions, their associated units of measurement, and cardinalities.

2. Automated ingestion and pre-processing. The completed Excel files are processed using a custom Python script that extracts the structured content and converts it into standardized, machine-readable formats: CSV files for vocabularies of terms, and JSON files for the structural representation of experiments and simulations. Where needed, experts provide additional description for experiment and simulation attributes that will be used for ontology generation. These outputs serve as the basis for subsequent automated steps, including semantic mapping and ontology generation. At this stage, the data remain ontology-agnostic and do not include any references to external ontological resources.

3. LLM-assisted semantic mapping. In this automated stage, the methodology builds upon NeOn Scenario 3, which emphasizes the reuse of existing ontological resources, by incorporating large language model (LLM) capabilities into a custom Python workflow. A dedicated Python script ingests project-specific CSV files from the previous step containing attribute terms, associated experiments or simulations, and reported measurement units. The script then proceeds in two phases. First, deterministic lookups are performed against curated dictionaries: HMIO derived units are checked, while standard units are matched against the QUDT ontology by symbol or unit name. Second, when no direct match is found, the script invokes the OpenAI GPT-3.5 model through the openai. ChatCompletion API. The model is prompted to (i) propose a canonical form of the reported unit (e.g. normalizing “microns” or “um” to “micrometer”), and (ii) select the most appropriate QUDT code from a shortlist of candidates generated by the dictionary search. This two-step use of the LLM ensures robust handling of noisy or heterogeneous input while constraining the output to valid ontology codes. Finally, identified QUDT units are cross-referenced against a mapping table to the EMMO. The result of this stage is an enriched set of CSV files in which each attribute is linked to HMIO, QUDT, and EMMO units. These enriched files constitute a draft vocabulary, providing both preliminary mappings and standardized definitions to be refined in the subsequent ontology engineering phase.

4. Iterative expert validation. The enriched CSV files are circulated to the domain experts in Excel format for systematic review. Experts evaluate the correctness of the proposed ontology alignments, revise term descriptions, and resolve any semantic ambiguities. Revisions are made directly within the structured CSV files to maintain traceability and ensure consistency with the automated pipeline. This human-in-the-loop process enables efficient refinement while ensuring that the resulting ontology accurately reflects domain-specific knowledge and usage. The validation cycle may be repeated multiple times, consistent with the iterative principles of the NeOn methodology.

5. Formal ontology generation and refinement. Following expert validation, the finalized CSV and JSON files serve as structured, non-ontological resources that are transformed into a formal ontology. This step corresponds to NeOn Scenario 8, which involves restructuring domain knowledge into a coherent and reusable ontological model. A Python script automates this process by generating an OWL ontology (in Turtle syntax), incorporating class hierarchies, object and data properties, and logical constraints. The ontology explicitly represents experimental and simulation processes using EMMO-aligned concepts such as Simulation, Experiment, hasInput, and hasOutput, ensuring semantic clarity and consistency across workflows.

6. Publication and dissemination. The final ontology is published through a persistent URL (purl.org), ensuring it is both findable and citable by the broader scientific community (HyWay Consortium, 2025). This step supports long-term accessibility and facilitates reuse in other projects or domains.

### 2.3 Integration architecture of the Data and Knowledge Management Platform (DKMP)

This section outlines the architectural design for integrating the Hydrogen-Material Interaction Ontology (HMIO) into the Digital Knowledge Management Platform (DKMP). The focus is on the modular, ontology-driven architecture that enables semantically structured and interoperable data management. Within this architecture, HMIO plays a central role in ensuring that all data entered, stored, and retrieved is aligned with FAIR data principles from the point of creation.

**Figure 2:**
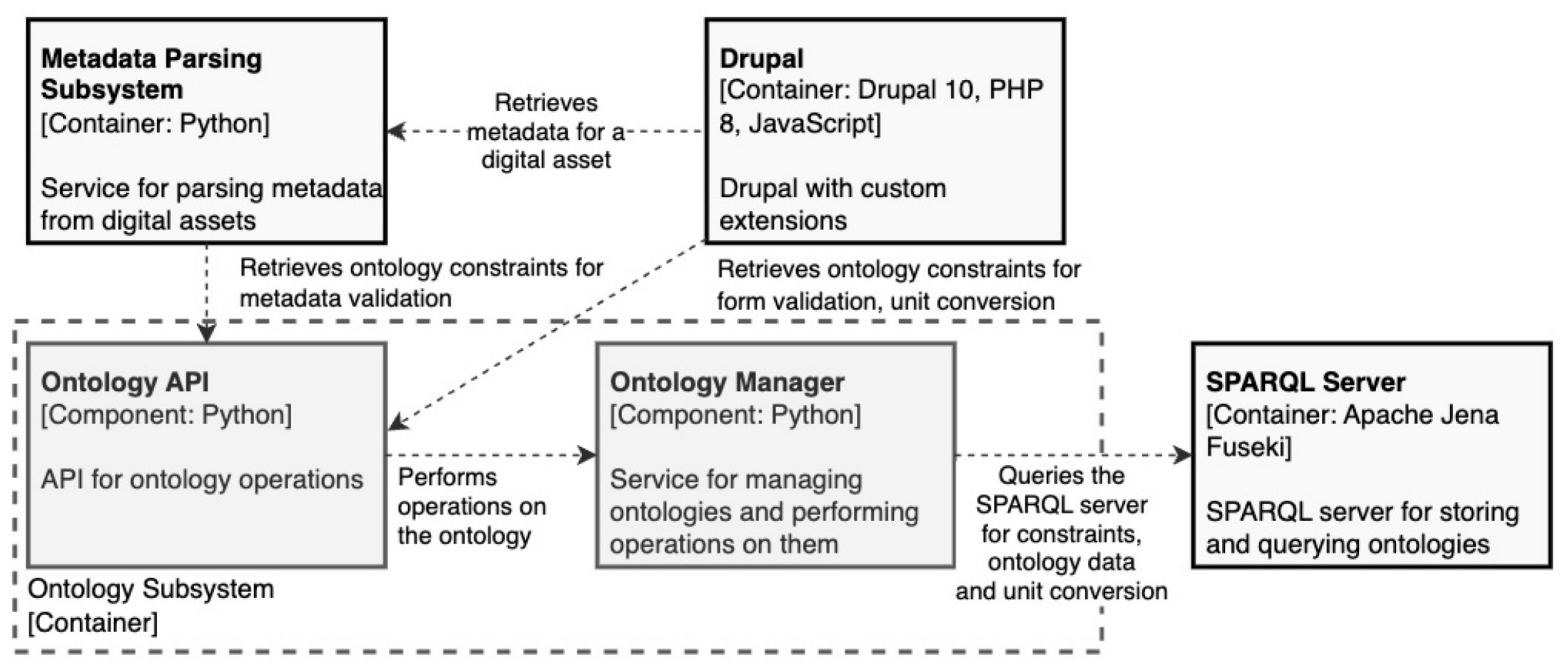
Drupal integration with ontology subsystem.

The platform is implemented using a modular, service-oriented architecture. Central to this architecture is the Ontology Subsystem (see Figure 2), comprising a Python-based service layer and a dedicated Apache Jena Fuseki SPARQL endpoint. The subsystem manages HMIO alongside external ontologies such as the Elementary Multiperspective Material Ontology (EMMO) and the Quantities, Units, Dimensions, and Data Types (QUDT) ontology. These ontologies are represented in RDF and exposed via SPARQL queries, allowing other platform components to access ontological definitions, semantic constraints, and unit specifications dynamically.

A key integration point is the Drupal (an open source content management framework) based front-end, which interacts with the Ontology Subsystem to enable ontology-driven user experiences. When a user initiates the creation or editing of an entity-such as an experiment or simulation, the front end queries HMIO to retrieve the relevant ontological class and its associated properties, cardinality constraints and expected data types, quantity and unit information are derived from QUDT. The retrieved information is used to dynamically build user interfaces, such as data entry forms that enforce correct input types, semantic relationships, and unit consistency. Additionally, the platform features a unit conversion widget that allows users to input measurements, switch between compatible units, and automatically converts values using QUDT conversion coefficients.

This tight integration of ontology services ensures that all data captured through the front end is semantically valid at the point of entry, conforming immediately to the HMIO schema. As a result, the platform avoids the need for *post-hoc* metadata alignment or semantic cleaning-common challenges in traditional data management systems-and guarantees compliance with FAIR principles throughout the data lifecycle.

**Figure 3:**
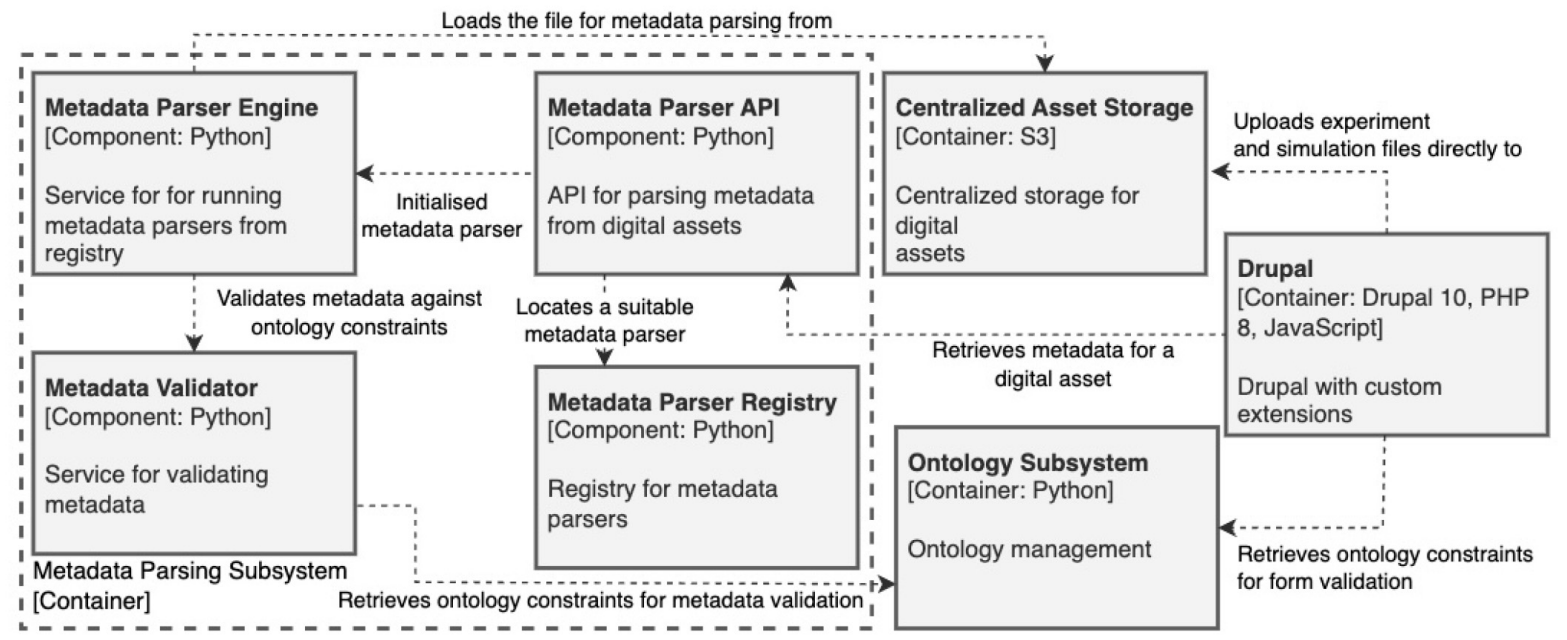
Metadata parsing subsystem.

The architecture also incorporates a Metadata parsing subsystem (see Figure 3), which processes incoming datasets from modeling workflows and experiments. This subsystem extracts metadata from the provided files, aligns it with the HMIO using specialized wrappers and the Ontology Subsystem, and ensures semantic consistency before ingestion into the platform's knowledge base. In case the necessary data cannot be extracted from the provided input files, the user needs to provide it manually.

**Figure 4:**
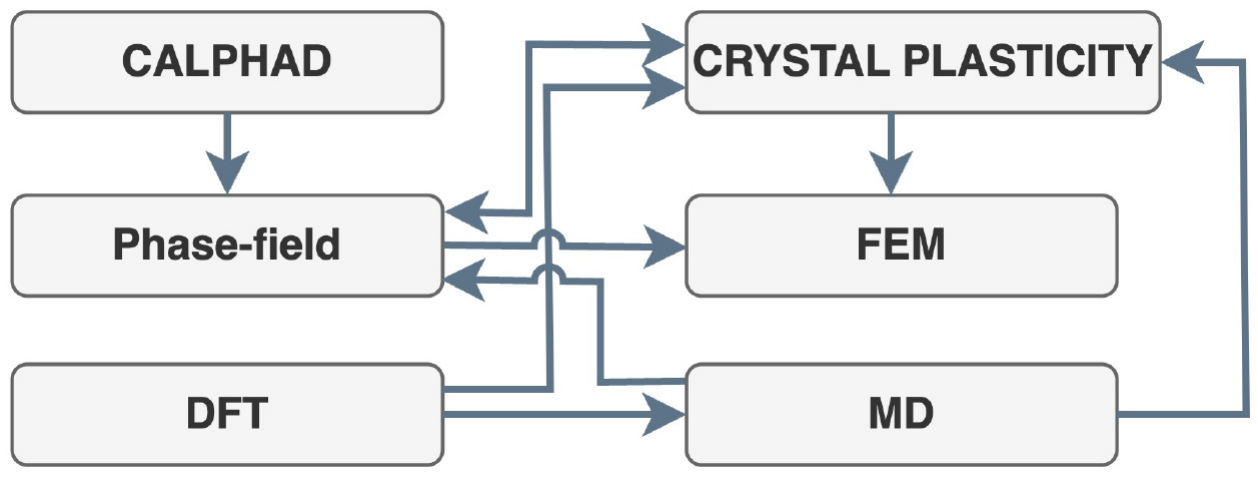
Integration between modeling suites and techniques.

DKMP is integrated with Modularised Multiscale and Multiphysics Materials Modeling Platform (ModularMMM), which enables the semantic integration of various simulation tools and techniques used in the HyWay project. ModularMMM (see Figure 4) coordinates data exchange between models operating at different scales-from atomistic simulations (e.g., Density Functional Theory, Molecular Dynamics) to continuum-level finite element models (e.g., ABAQUS). Each modeling tool and its modeling technique is encapsulated by a semantic wrapper that transforms native inputs and outputs into a representation aligned with HMIO. This approach supports automatic interoperability between tools, ensuring that simulation results remain semantically consistent across workflows. These data are automatically annotated and converted by ModularMMM's wrappers, enabling efficient reuse in downstream simulations without manual data translation. This architecture ensures semantic traceability of parameters across modeling stages.

## 3 Results

The application of the described methodology and technical architecture yielded several key results, which serve as a validation of the approach. These include the development of a comprehensive domain ontology, the successful implementation of an ontology-driven data management platform, and an evaluation of the overall system.

### 3.1 The hydrogen-material interaction ontology (HMIO)

One of the principal scientific outputs of this work is HMIO, a domain-specific ontology developed as a result of applying the collaborative, LLM-supported methodology introduced in this study. HMIO serves as the semantic backbone of the HyWay project and plays a central role in enabling ontology-driven data management within the DKMP. Together, the development methodology, the resulting ontology, and its architectural integration form a comprehensive approach to achieving FAIR-compliant, interoperable workflows across experimental and simulation domains.

The application of the methodology has resulted in an ontology with substantial initial domain coverage. The current version of HMIO formally models metadata structures for 29 experimental methodologies and 14 simulation model types relevant to the study of hydrogen-material interactions, ranging from atomistic-scale simulations to macroscopic experimental techniques. As part of an iterative development approach, HMIO will continue to be expanded and refined throughout the project to accommodate additional workflows, concepts, and domain requirements as they emerge.

HMIO is designed with a modular architecture to promote clarity, maintainability, and extensibility. The ontology consists of several interlinked modules, each focused on a specific aspect of the hydrogen-materials domain. The central file, hmio.ttl, serves as the entry point, providing the namespace declaration and importing all domain-relevant components. Experimental concepts and workflows are defined in experiment_terms.ttl and experiments.ttl, while simulation-related definitions are specified in simulation_terms.ttl and simulations.ttl. In addition, units.ttl extends the QUDT ontology with domain-specific derived units to enable consistent and interoperable representation of physical quantities, including composite units such as millinewton per second.

To ensure semantic interoperability beyond the scope of the project, HMIO is formally aligned with well-established reference ontologies. Core concepts in materials science are mapped to the EMMO, while all quantities, units, dimensions, and data types are standardized using the QUDT ontology. This alignment guarantees that data annotated with HMIO remains compatible with broader scientific semantic infrastructures and enables cross-domain reuse.

### 3.2 Realization of ontology-driven data management in DKMP

This section presents the implemented functionality of the DKMP, demonstrating how the HMIO is utilized to support ontology-driven data entry and validation. Building on the modular architecture described in Section 2.3, the platform showcases practical integration of semantic technologies for the structured and interoperable collection of experimental and simulation metadata.

**Figure 5:**
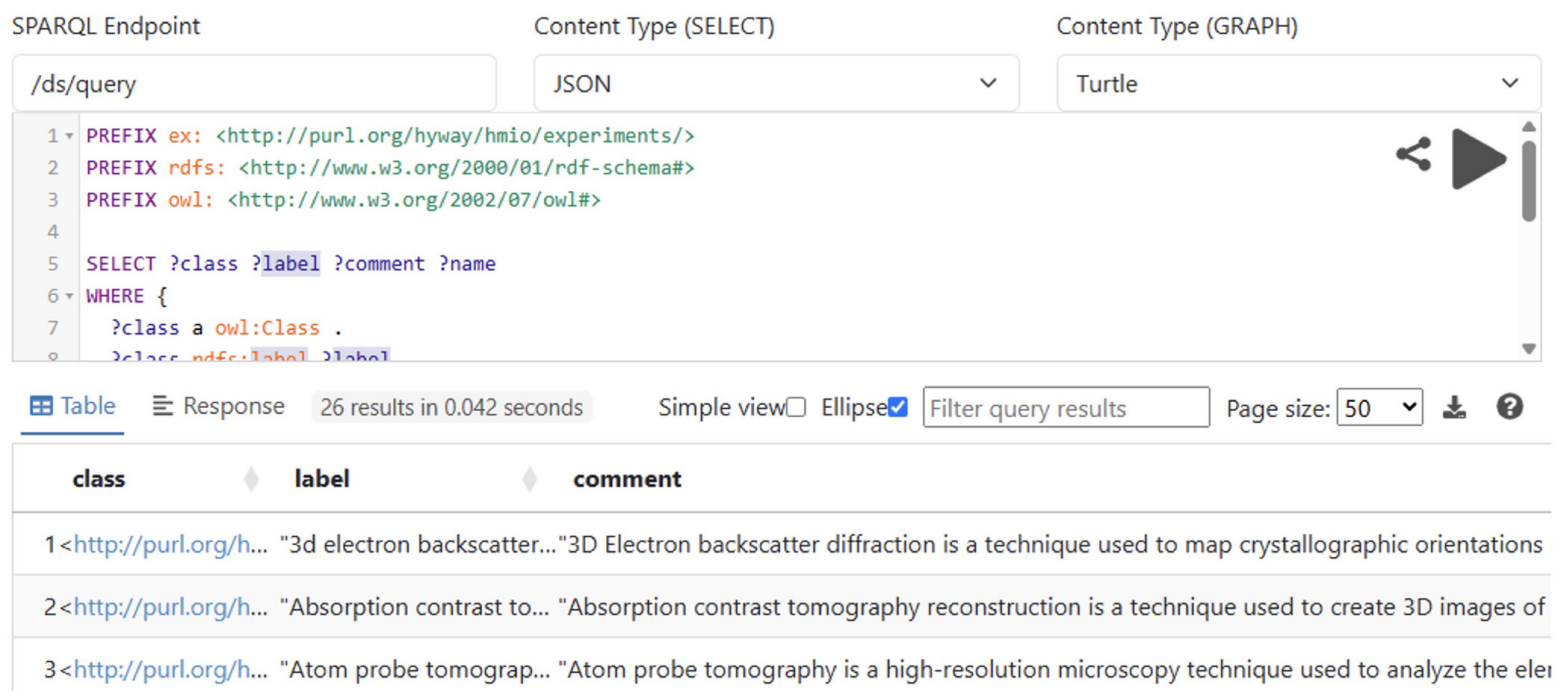
SPARQL query to retrieve all experiments from the ontology.

The system enables the automatic generation of Drupal content types and web forms based directly on schema definitions contained in HMIO. A Python-based Ontology subsystem processes the OWL ontology files hosted in a version-controlled GitLab repository, resolves imported dependencies-including QUDT and EMMO-and loads the ontology data into an Apache Jena Fuseki triple store. The ontologies are then queried using SPARQL to extract classes, properties, and associated metadata that describe experiments and simulations (see example query in Figure 5).

**Figure 6:**
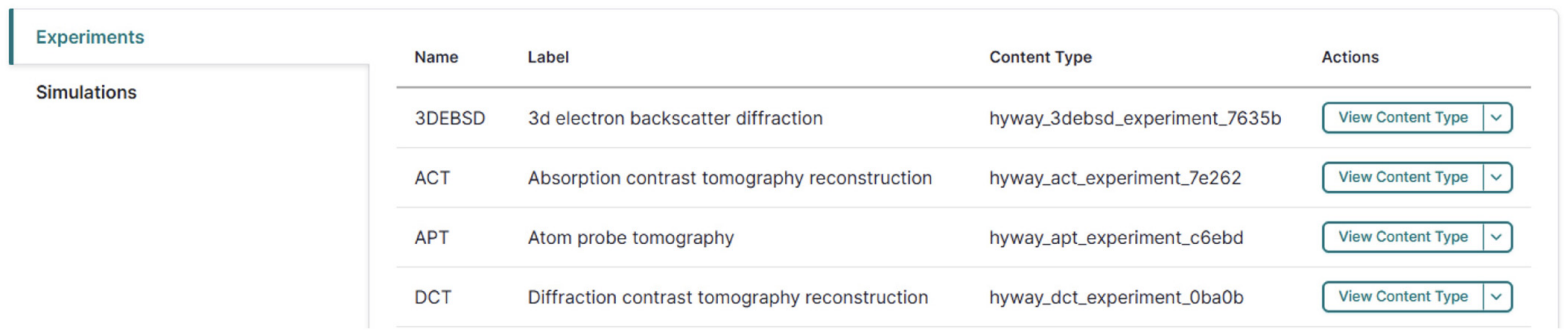
Ontology-driven Drupal content type generation.

A custom Drupal module connects it with the Ontology subsystem and consumes this semantic information to dynamically construct content types and their input forms in accordance with the ontology (see Figure 6). Each form field corresponds to an ontological property, ensuring that constraints on data type, cardinality, and semantic relationships are preserved. As a result, data captured via the user interface is immediately aligned with the underlying ontology, reducing the need for manual schema configuration and eliminating *post-hoc* data cleaning.

**Figure 7:**
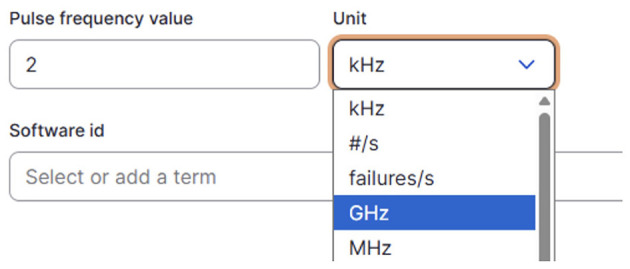
QUDT-based unit entry widget.

The platform also provides a custom unit entry widget (see Figure 7) using the references established between HMIO and QUDT ontology. During data entry, users are provided with an appropriate unit selection that reflects allowed values from the ontology, and the system performs automatic unit conversions between compatible units using QUDT's conversion factors. This ensures consistency in the representation of physical quantities and supports semantic interoperability across experiments and simulations.

The realization of this ontology-based infrastructure confirms the feasibility of deploying semantic data management workflows within scientific platforms. The implementation enables researchers to capture FAIR-compliant metadata through an intuitive interface, backed by semantic validation and ontology-driven logic.

### 3.3 Validation and evaluation of the approach

The proposed methodology and architectural ontology integration pattern were validated through the development of prototypes and iterative evaluation by domain experts. A key quantitative outcome is the scope and coverage of the HMIO, which formally represents metadata for 29 experimental methodologies and 14 simulation model types relevant to hydrogen-material interaction studies. This level of coverage demonstrates the methodology's capability to support complex scientific workflows. The combined use of LLMs and expert-driven validation proved highly effective in accelerating ontology development without compromising semantic accuracy. Project partners involved in expert review cycles confirmed that the methodology supported structured knowledge elicitation and reduced the manual effort traditionally associated with ontology engineering.

Furthermore, core components of the DKMP were successfully implemented. In addition to the ontology integration services, the platform includes authentication and authorization via Keycloak and support for resumable uploads of large files using S3-compatible horizontally scalable object storage. These components were deployed and tested to validate the technical feasibility of the proposed architecture. The web-based data entry interface for experiments and simulations demonstrates full end-to-end integration and serves as a proof of concept for the platform's ability to semantically generate user interfaces. This ensures that input data conform to ontology-defined constraints at the point of capture, realizing a FAIR-by-design data management approach.

## 4 Discussion

The presented results demonstrate the feasibility and effectiveness of the proposed methodology for collaborative LLM supported ontology development and its integration into a semantic data management platform. This section discusses the implications of the findings, compares the work with previous research, and critically evaluates the strengths and limitations of the approach, as well as future directions.

The success of the HyWay methodology is attributed to its combination of large language model (LLM) assisted automation and expert-driven validation. This hybrid approach addresses long-standing challenges in ontology engineering, including the labor-intensive nature of manual modeling and the complexity of eliciting structured knowledge from domain experts. LLMs accelerated the development process by mapping domain-specific terms, extracted from structured Excel surveys, to established ontologies such as EMMO and QUDT. This automation significantly reduced the effort required from experts, enabling them to focus on the semantic quality and coherence of the ontology.

At the same time, the iterative expert validation process was essential. While the large language models demonstrated a reasonable grasp of general experimental methodologies, they exhibited limitations when interpreting the specific modeling techniques used in domain-specialized tools such as DAMASK. Experts played a critical role in identifying and correcting these mismatches, resolving semantic ambiguities, and refining conceptual definitions. This collaborative process not only improved the accuracy and domain alignment of the ontology but also clarified and formalized expert knowledge through structured discussion. The integration of rapid, machine-assisted mapping with rigorous human validation proved to be a reliable and efficient strategy for developing a semantically rich and technically sound ontology.

Compared with prior research, the HyWay methodology represents a pragmatic evolution of existing ontology development frameworks. It builds on the NeOn methodology, particularly Scenario 3, which focuses on reusing existing ontological resources. Unlike approaches such as UPON Lite or MeLOn, which emphasize collaborative workflows, HyWay incorporates automation directly into the knowledge acquisition and alignment stages. It differs from typical LLM-based ontology learning, which often relies on unstructured text corpora, by instead leveraging structured input from domain experts. The role of the LLM is limited to semantic suggestion and mapping, rather than full ontology generation. This enables a balanced system where LLMs support, but do not replace, human reasoning.

The approach is tightly coupled with the architecture of the DKMP. The automatic generation of ontology-driven data entry forms illustrates the platform's ability to ensure semantic validity at the point of data collection. This capability helps to enforce FAIR principles by design, preventing issues related to data quality and interoperability from arising later in the data lifecycle.

Key strengths of the methodology include its scalability, alignment with semantic data management principles, and its capacity to meaningfully engage experts in a validation-oriented role. However, several limitations must be acknowledged. The effectiveness of the semantic mapping is dependent on the clarity and completeness of the initial survey inputs. Poorly structured or ambiguous data increases the burden on the validation phase. Additionally, the internal mechanisms of LLMs remain opaque, necessitating critical human oversight to mitigate hallucinations and unintended biases.

One of the significant challenges for future work is managing the evolution of the HMIO. As the ontology expands and evolves, maintaining compatibility with previously recorded metadata instances becomes increasingly difficult. Addressing this issue will require structured versioning, automated data migration scripts, and mechanisms for version-aware querying within DKMP.

Further research should explore the fine-tuning of domain-specific language models trained on curated materials science datasets, with the goal of improving semantic precision and reducing reliance on generic models. Enhancing the interactivity and usability of validation interfaces would also support more effective expert feedback. Lastly, the methodology could be adapted to other complex scientific domains, such as life sciences or environmental science, to validate its generalisability and broader applicability.

## 5 Conclusion

This paper has presented the design, implementation, and validation of a novel methodology that extends established ontology engineering principles through the incorporation of LLM-driven automation. By combining the efficiency of LLM-assisted semantic mapping with the rigor of an iterative, expert-in-the-loop validation framework grounded in the NeOn methodology, the approach effectively addresses key limitations of traditional ontology development. The resulting ontology, and its integration into the DKMP, demonstrate the viability of this methodology and the proposed architectural integration pattern in a complex, real-world scientific domain. The platform's dynamic use of the ontology to drive interface generation ensures semantic consistency and high-quality data capture. Taken together, these contributions offer a practical and scalable blueprint for the development of ontology-driven data infrastructures in materials science and related fields.

This paper has presented the design, implementation, and validation of a novel methodology that extends established ontology engineering principles through the incorporation of LLM-driven automation. Specifically, the OpenAI GPT-3.5 model was integrated into a Python-based workflow to support semantic mapping of experimental and simulation attributes against reference ontologies, particularly QUDT and EMMO. By combining deterministic dictionary lookups with LLM-assisted canonicalisation and code selection, the approach achieved robust alignment of heterogeneous input data with standardized ontology terms.

Building on the NeOn methodology, particularly Scenario 3 for reuse of existing ontological resources, the workflow demonstrated how LLMs can accelerate knowledge acquisition while domain experts ensure semantic accuracy through iterative validation. The resulting Hydrogen-Material Interaction Ontology (HMIO) formalizes metadata structures for 29 experimental methods and 14 simulation types, providing broad coverage of hydrogen-materials interaction studies. These results provide both a validated methodology and a working technical implementation that illustrate the feasibility and scalability of LLM-supported collaborative ontology engineering.

A key result is the successful integration of HMIO into the Data and Knowledge Management Platform (DKMP). Here, the ontology is dynamically queried to generate ontology-driven user interfaces, such as data entry forms that enforce correct cardinalities, input types, and unit consistency. Thus, what was previously described as “the platform's dynamic use of the ontology” refers concretely to the automatic construction of FAIR-by-design data management workflows, where semantic validation occurs directly at the point of data entry.

Looking ahead, several challenges remain open. These include ensuring version management of HMIO as it evolves, improving interoperability with external datasets, and mitigating the limitations of general-purpose LLMs through fine-tuning on domain-specific corpora. A particular challenge is the definition of derived units of measure, which are frequently required in scientific workflows but are not always present in existing ontologies such as QUDT. Here, LLMs provide an opportunity to automatically generate and normalize such derived units, ensuring consistency with both expert-curated HMIO extensions and reference ontologies. Future work will also explore extending the methodology to additional scientific domains, as well as developing user-friendly interfaces for expert validation.

## Data Availability

The original contributions presented in the study are included in the article/supplementary material, further inquiries can be directed to the corresponding author.
